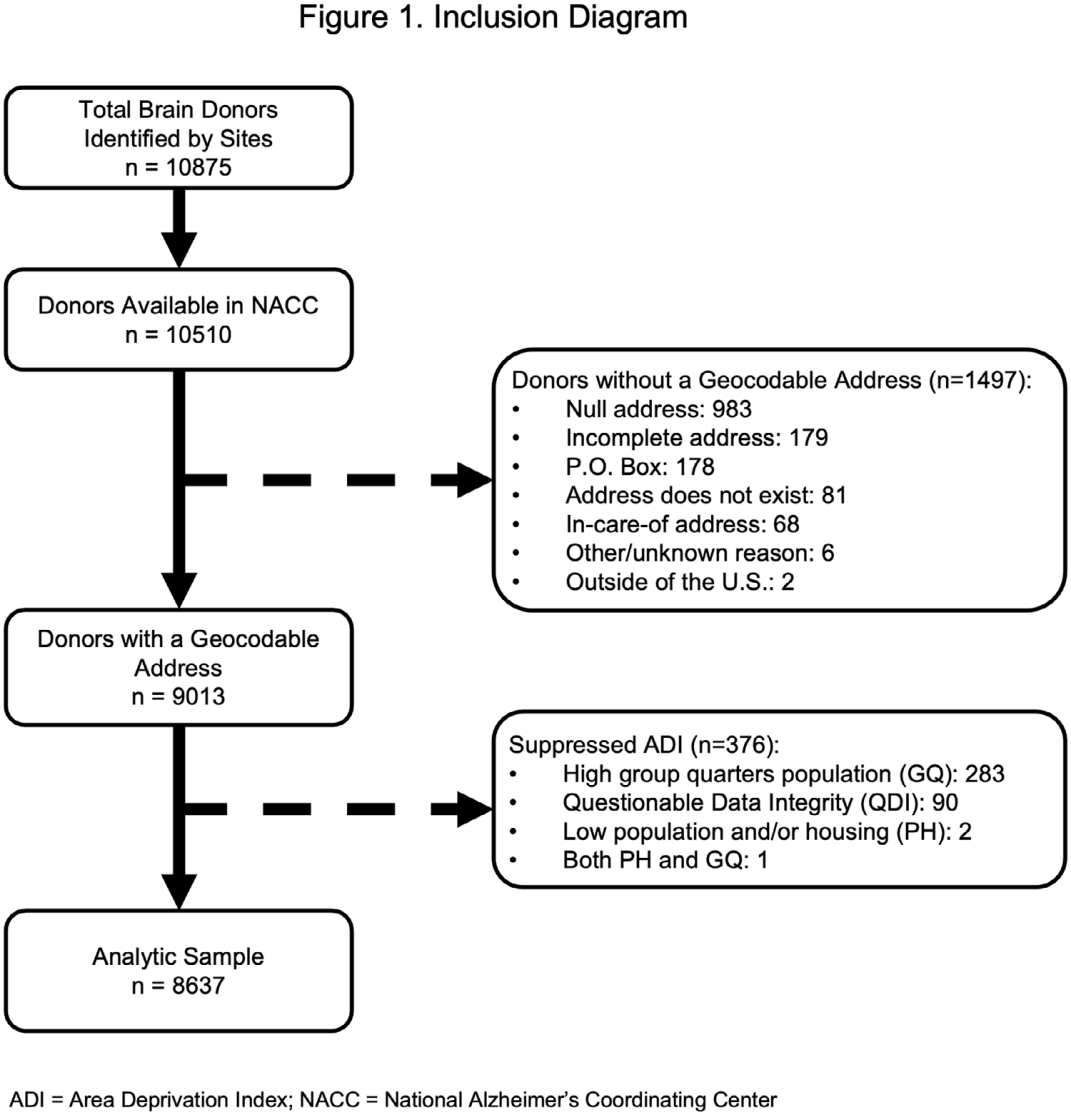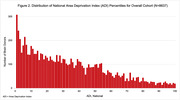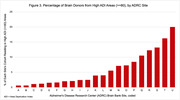# Over‐Representation of Extremely Wealthy Neighborhood Social Exposomes for Brain Donors within Alzheimer’s Disease Research Center Brain Banks assessed by the Neighborhoods Study

**DOI:** 10.1002/alz.087530

**Published:** 2025-01-09

**Authors:** Amy J.H. Kind, Barbara B. Bendlin, W. Ryan Powell, Amanda DeWitt, Yixuan Cheng, Luke Chamberlain, Jessica Sharrow, Brittney Lyons Boone, Erin L. Abner, Michael L. Alosco, Liana G. Apostolova, Kelly M. Bakulski, Lisa L. Barnes, James R. Bateman, Thomas G. Beach, David A. Bennett, James B. Brewer, Carmen Carrion, Joshua Chodosh, Suzanne Craft, Raina Croff, Anthony Fabio, Sarah Tomaszewski Farias, Felicia Goldstein, Victor W. Henderson, Thomas K Karikari, Julia Kofler, Anna M. Kucharska‐Newton, Melissa Lamar, Serggio Lanata, Rebecca J Lepping, Jennifer H Lingler, Samuel N. Lockhart, Jonathan D Mahnken, Karyn Marsh, Oanh L. Meyer, Bruce L. Miller, Jill K Morris, Judith A. Neugroschl, Maureen K. O'Connor, Henry L Paulson, Richard J. Perrin, Corinne Pettigrew, Aimee Pierce, Cyrus A. Raji, Eric M. Reiman, Shannon L. Risacher, Robert A Rissman, Patricia Rodriguez Espinoza, Mary Sano, Andrew J. Saykin, Geidy E Serrano, Anja Soldan, David L Sultzer, Rachel A. Whitmer, Thomas Wisniewski, Randall Woltjer, Carolyn W. Zhu

**Affiliations:** ^1^ Center for Health Disparities Research, University of Wisconsin School of Medicine and Public Health, Madison, WI USA; ^2^ Sanders‐Brown Center on Aging, University of Kentucky, Lexington, KY USA; ^3^ Boston University School of Medicine, Boston, MA USA; ^4^ Indiana University School of Medicine, Indianapolis, IN USA; ^5^ University of Michigan School of Public Health, Ann Arbor, MI USA; ^6^ Rush University Medical Center, Chicago, IL USA; ^7^ Wake Forest University School of Medicine, Winston‐Salem, NC USA; ^8^ Civin Laboratory for Neuropathology, Banner Sun Health Research Institute, 10515 W Santa Fe Drive, Sun City, AZ 85351, Sun City, AZ USA; ^9^ University of California, San Diego, La Jolla, CA USA; ^10^ Yale University, New Haven, CT USA; ^11^ NYU Alzheimer’s Disease Research Center, New York, NY USA; ^12^ Oregon Health & Science University, Portland, OR USA; ^13^ University of Pittsburgh, Pittsburgh, PA USA; ^14^ University of California‐ Davis, Sacramento, CA USA; ^15^ Emory University School of Medicine, Atlanta, GA USA; ^16^ Stanford University School of Medicine, Stanford, CA USA; ^17^ University of North Carolina Gillings School of Global Public Health, Chapel Hill, NC USA; ^18^ Memory and Aging Center, UCSF Weill Institute for Neurosciences, University of California, San Francisco, San Francisco, CA USA; ^19^ University of Kansas Medical Center, Kansas City, KS USA; ^20^ Wake Forest School of Medicine, Winston‐Salem, NC USA; ^21^ NYU Grossman School of Medicine, New York, NY USA; ^22^ University of California, Davis School of Medicine, Sacramento, CA USA; ^23^ Memory & Aging Center, Department of Neurology, University of California in San Francisco, San Francisco, CA USA; ^24^ Icahn School of Medicine at Mount Sinai, New York, NY USA; ^25^ University of Michigan, Ann Arbor, MI USA; ^26^ Washington University in St. Louis, St. Louis, MO USA; ^27^ Johns Hopkins University School of Medicine, Baltimore, MD USA; ^28^ Mallinckrodt Institute of Radiology, Washington University, St. Louis, MO USA; ^29^ Banner Alzheimer’s Institute, Phoenix, AZ USA; ^30^ University of Southern California, San Diego, CA USA; ^31^ Stanford University, Palo Alto, CA USA; ^32^ Mount Sinai School of Medicine, New York, NY USA; ^33^ Indiana University, Indianapolis, IN USA; ^34^ University of California, Irvine, Irvine, CA USA; ^35^ University of California, Davis, Davis, CA USA; ^36^ New York University Grossman School of Medicine, New York, NY USA; ^37^ NIA‐Layton Oregon Alzheimer’s Disease Research Center, Oregon Health & Science University, Portland, OR USA; ^38^ Icahn School of Medicine, Mount Sinai Hospital, New York, NY USA

## Abstract

**Background:**

Adverse social exposome (indexed by national Area Deprivation Index [ADI] 80‐100 or ‘high ADI’) is linked to structural inequities and increased risk of Alzheimer’s disease neuropathology. Twenty percent of the US population resides within high ADI areas, predominantly in inner cities, tribal reservations and rural areas. The percentage of brain donors from high ADI areas within the Alzheimer’s Disease Research Center (ADRC) brain bank system is unknown.

**Objective:**

Determine ADI for brain donors from 21 ADRC sites as part of the on‐going Neighborhoods Study.

**Methods:**

All brain donors in participating ADRC sites with NACC neuropathology data and personal identifiers for ADI linkage (N = 8,637) were included (Figure 1). Geocoded donor addresses were linked to time‐concordant ADI percentiles for year of death.

**Results:**

Overall, only 5.6% of ADRC brain donors (N = 488) resided in a high ADI (disadvantaged) neighborhood at death. The remaining donors resided in more advantaged neighborhoods, with nearly 40% of donors living in the wealthiest quintile of neighborhoods, and over 300 brain donors originating from the wealthiest 1% of US neighborhoods (Figure 2). Donors from high ADI (disadvantaged) neighborhoods identified as 87% White (n = 424), 11% Black (55), 1% Multiracial (6) and <1% other/unknown race (3), with 1% Hispanic (5). None identified as American Indian/Alaska Native or Native Hawaiian/Pacific Islander/Asian. In comparison, donors from low ADI neighborhoods were 94% White (n = 7680), 3% Black (273), 1% Multiracial (75), <1% American Indian/Alaska Native (11), <1% Native Hawaiian/Pacific Islander/Asian (60), and <1% other/unknown race (50), with 3% Hispanic (230). Sex distribution was similar (54%, 51% female, respectively). Inclusion of high ADI donors varied dramatically across the 21 ADRC brain banks from a low of 0.6% to high of 20% of all a site’s donors (Figure 3).

**Conclusions:**

ADI was determined for over 8,600 brain donors in the ADRC system, demonstrating a marked over‐representation of donors from very low ADI (extremely wealthy) neighborhoods, in addition to site‐to‐site variability. This is the first time a comprehensive cross‐sectional social exposome assessment of this nature has been performed, opening windows for additional mechanistic study of the social exposome on brain pathology. Life course ADI assessments are on‐going.